# Rituximab for the treatment of refractory pediatric autoimmune diseases: a case series

**DOI:** 10.4076/1757-1626-2-6609

**Published:** 2009-08-06

**Authors:** Nikolay Tzaribachev, Ina Koetter, Jasmin B Kuemmerle-Deschner, Joerg Schedel

**Affiliations:** 1 University Children's Hospital, Hoppe-Seyler Str. 1, 72076, Tuebingen, Germany; 2 University Hospital, Department of Internal Medicine II, Otfried-Mueller-Str. 10, 72076, Tuebingen, Germany

## Abstract

**Introduction:**

To report on efficacy, tolerability and safety of rituximab in children with refractory autoimmune diseases.

**Case presentation:**

Five patients (juvenile dermatomyositis, Wegener's granulomatosis, systemic lupus erythematosus, myasthenia gravis and multiple sclerosis with systemic lupus erythematosus) were treated with rituximab and followed for a median time of 2.5 years. Two patients achieved remission (systemic lupus erythematosus, Wegener's granulomatosis). Three patients had a refractory disease course and underwent autologous stem cell transplantation. Of those, two achieved remission (juvenile dermatomyositis, myasthenia gravis), one died of complications after transplantation (multiple sclerosis/systemic lupus erythematosus). No severe adverse events occurred.

**Conclusion:**

Efficacy of rituximab was variable ranging from complete remission to inefficacy. Treatment was safe.

## Introduction

B cells are considered to be major players in the pathogenesis of most autoimmune diseases. B cells produce antibodies and cytokines and are involved in antigen presentation [1,2]. The chimeric anti-CD-20 antibody (rituximab [RTX]) leads to depletion of most peripheral B-cells and has demonstrated a good efficacy and safety profile in patients with rheumatoid arthritis (RA) in randomized, double-blind, placebo controlled trials [3]. RTX is approved in the USA and most European countries for the treatment of RA after inefficacy of one TNFα-blocking agent.

With regards to pediatric patients no randomized, placebo controlled trials with RTX have been published so far. Most available data are based upon case reports or retrospective case series in a variety of different diseases with a small number of patients [3-9].

Here we report our experience on efficacy, tolerability and safety in five pediatric patients treated with RTX for refractory autoimmune diseases at our institution.

Informed consent was obtained from all patients and their parents prior to treatment. Clinical and laboratory data were evaluated retrospectively by chart review. The retrospective data acquisition was approved by the local Ethics Committee of the University of Tuebingen.

## Case presentations

Patient's demographic and baseline characteristics are summarized in Table 1.

**Table 1:** Patients' characteristics and concomitant medication

Patient/Gender	Diagnosis	Age at Disease Onset [years]	Disease Symptoms	Disease Duration before Treatment [years]	Medication before Rituximab treatment	Rituximab Indication	Rituximab Dosis [mg/m]^2^	Rituximab Courses [4 doses/course]	Time to Return of peripheral B-cells^3^	Time to Relapse	AE/SAE	Follow-up Time [years]
1. female	JDM	14	heliotropic rash Gottron's patcnes muscul. weakness contractures difficult breathing difficult swallowing diffjcult speach	1	MP(10 pulses) P MTX IVIG (10 pulses) CSA	progressive disease despite therapy	375	2	*11 months **progress without B-cells	*3 months **3 months	none/none	2
2. male	WG	10	episcleritis arthralgia cough skin ulcers fever weight loss	1	MP (2 pulses) CYC (6 pulses) P MTX AZA	relapsing disease despite therapy	375	1	5 months	no relapse	none/none	2
3. male	SLE	7	malar rash glomerulonephrilis CNS vasculitis	2	AZA MP (? pulses) CYC plasmapheresis	severe CNS involvement	375	1 single dose	no data	no relapse	none/none	6
4. female	SLE WG	13 for SLE 14 for MG	thrombosis (DVTJ fever sweating fatigue weight loss malar rash loss of strength diplopic images	4	P, AZA CYC (6 pulses) MP (6 pulses) MMF Pyridostigmine plasmapheresis^1^ NSAlDs	*relapsing MG despite therapy **disease flare before ASCT ***disease flare after ASCT	375	3	*6 months **no B-ceii retum[dis.flare without B-cells) ***2 months	*response clinically insignificant **no response followed by ASCT ***no relapse	mild bronchitis/none	1.5
5. female	MS SLE	3 for MS 17 for SLE	opticus neuritis hemiparesis malar rash arthritis	1.5	MP (multiple pulses) intraocular Steroids CYC (12 pulses) (β-lnterferon AZA MMF NSAlDs	*relapsing/progressive SLE nephritis despite therapy **autoimmune anemia/thrombo cytopenia after ASCT	375	2 2 single doses	*12 months (including time after ASCT) **no B-cell depletion after 2 single doses	*partial to nituximab but development of glomerulonephritis **partial response, repeatedly low erythrocyties/thrombocytes	none/none patient's death due to Evans syndrome/disease recurrence	1

JDM, juvenile dermatomyositis; WG, Wegener's granulomatosis; SLE, systemic lupus erythematodes; MG, myasthenia gravis; MS, multiple sclerosis; CNS, central nervous system; ASCT, autologous stem cell transplantation; MP, methylprednisolone (dosing regimen: 30 mg/kg-max. 1 g/day for 3 days); P, prednisolone (dosing regimen: 2 mg/kg/day); IVIG, iv immunoglobulins (dosing regimen: 1 g/kg/day for 3 days); CSA, cyclosporine A (dosing regimen: 3 mg/kg/day); MTX, methotrexate (iv-dosing regimen: 1 mg/kg/week; sc-dosing regimen: 15 mg/kg/week); AZA, azathioprine (dosing regimen: 3 mg/kg/day); CYC, cyclophosphamide (dosing regimen: iv 750 mg/m^2 ^/every 4 weeks); MMF, mycophenolate mofetil (dosing regimen 1 - 3 g/day-depending on MMF blood levels); NSAIDs, non-steroidal anti-inflammatory drugs; AE, adverse event; SAE, serious adverse event

*^1st ^rituximab course

**2^nd ^rituximab course

***3^rd ^rituximab course

^1 ^Rituximab was applied after the lymphoma protocol: 4 × 375 mg/m^2 ^(two of the patients received single doses as well)

^2 ^the second plasmapheresis could not be accomplished

^3 ^B-cells were measured by fluorescence activated cell sorting (FACS) analysis

Conditioning protocol prior to ASCT included: fludarabine 120 mg/m^2^, cyclophosphamide 120 mg/m^2 ^and muromonab-CD3-10 mg.

RTX was applied according to the lymphoma protocol - 375 mg/m^2 ^/week over 4 weeks. Some of the patients received also single RTX doses. Prior to every RTX infusion single intravenous doses of prednisolone (1 mg/kg), diphenhydrinate (2 mg total dose) and ranitidine (150 mg total dose) were administered.

### Case report 1

In 2003 the diagnosis of juvenile dermatomyositis (JDM) was made in a 14 year old Caucasian girl (German). She presented initially with typical JDM symptoms-heliotropic rash, Gottron's patches and loss of strength in the proximal muscle groups. The muscle enzymes- creatine kinase (CK) and aldolase (ALD)- were significantly elevated. The patient also showed a slight elevation of the inflammatory parameters erythrocyte sedimentation rate (ESR) and C-reactive protein (CRP). The treatment included: high doses of intravenous (i.v.) methylprednisolone (MP) (1 g/day for 3 days) and oral (p.o.) prednisolone (PREDN) (2 mg/kg/day), i.v. methotrexate (MTX) (1 mg/kg/week), i.v. immunoglobulins (IVIG) (1 g/kg/day for 3 days) and oral cyclosporine A (CSA) (3 mg/kg/day) (Table 1).

After two months of treatment CK and ALD normalized. Nevertheless muscle strength decreased progressively and after one year of treatment the girl was unable to move and also showed difficulties in breathing and was incapable to swallow hard food. At that time a slow but continuous elevation of CK and ALD was observed. Also a diffuse gadolinium enhancement on repeated whole-body magnetic resonance imaging scans (WBMRI) was documented. Due to the persistent/progressive disease course and considering the young age of the patient and also the potential side effects of the immanent treatment with cyclophosphamide (CYC) including ovarial suppression by gonadotropin releasing hormone agonists, the indication for RTX was given.

**Figure 1:**
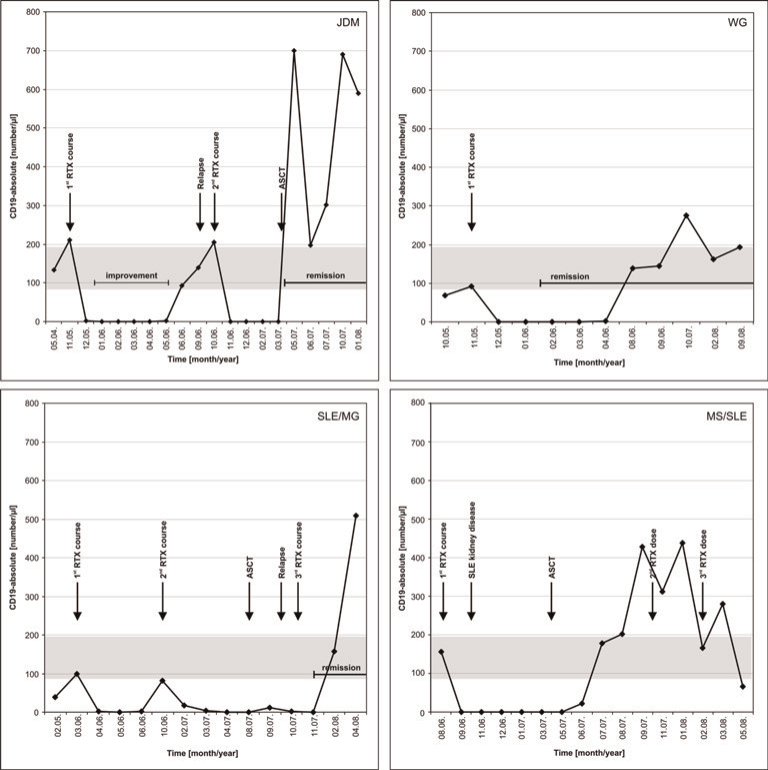
**B-cell depletion in patients 1 (JDM), 2 (WG), 4 (SLE/MG) and 5 (MS/SLE)**. No clear correlation between B-cell recurrence and disease flares can be seen. In patient 4 (SLE/MG) B-cells were reduced below normal levels due to treatment with cyclophosphamide. JDM, juvenile dermatomyositis; WG, Wegener's granulomatosis; SLE, systemic lupus erythematodes; MG, myasthenia gravis; MS, multiple sclerosis; ASCT, autologous stem cell transplantation; RTX, rituximab.

Together with MP pulses (1 g/kg/day for 3 days), four doses (1 course) of RTX were administered and the concomitant medication with MTX (20 mg/m^2 ^/week subcutaneously) was continued (Table 1, Figure 1). CK and ALD normalized promptly. Muscle strength also improved to a little degree. Improvement lasted for a total of six months. Immediately thereafter muscle strength deteriorated again, CK and ALD were again elevated and the patient received a second RTX course together with MP pulses (1 g/day for 3 days) and subcutaneous MTX (20 mg/m^2 ^/week) (Table 1, Figure 1). CK and ALD normalized over the period of two months. However, the muscle strength did not recover and the patient was severely disabled. Due to this unsatisfying response to RTX and the prominent myositic changes on WB-MRI autologous stem cell transplantation (ASCT) was performed, leading to complete drug free remission for 2.5 years by now.

### Case report 2

In 2004 a ten year old Caucasian boy (German) was diagnosed with Wegener's granulomatosis. He was treated for episcleritis with local antibiotics over the period of three months and developed thereafter recurrent fever attacks and a cough resistant to oral antibiotics. He also complained of perioral skin ulcers, knee- and hiparthralgia as well as an unexplained weight loss of 10 kg. The chest X-ray showed peribronchial infiltrates and patchy opacities in both upper lung fields and the CT-scan revealed peribronchial, contrast enhanced hyperdense areas. Laboratory investigation showed a high ESR, CRP and elevated proteinase-3 anti-neutrophil cytoplasmatic antibodies (c-ANCA). Lung biopsy revealed granulomatous, partially necrotic infiltrates. Although there were no clear vasculitic signs this result together with the clinical presentation and the elevated c-ANCA supported the diagnosis of Wegener's granulomatosis. Other organs were not involved. Treatment was started with i.v. MP (1 g/day for 3 days), p.o. PREDN (2 mg/kg/day), i.v. CYC (750 mg/m^2 ^/every 4 weeks for 6 months) and p.o. azathioprine (AZA) (3 mg/kg/day) (Table 1). Within eight weeks after treatment initiation ESR and CRP reached normal levels (low levels of c-ANCA persisted), skin changes disappeared and chest X-ray showed a normal result. Due to the clinical remission of the disease a slow tapering of the oral steroids was started. Only two weeks after discontinuing treatment with p.o. PREDN disease flared, evidenced by reoccurrence of cough, perioral and perianal skin ulcers and also elevated CRP and ESR. Interestingly, at that time the chest X-ray was normal. In order to confirm or rule out lung involvement chest magnetic resonance imaging (MRI) was used, which revealed peribronchial hyperintense lesions with gadolinium enhancement throughout both lungs. The young age and the potential long-term side effects of multiple CYC treatments in mind, one RTX course was administered (Table 1, Figure 1). The concomitant medication with AZA was continued and oral PREDN (2 mg/kg/day) was used as a bridging drug. This approach led to a resolution of symptoms within 4 weeks. PREDN was tapered over another 4 weeks, AZA was discontinued and remission was sustained by mycophenolate mofetil (MMF; 1-2 g/day depending on MMF blood levels; attempted: 2.5-3.5 μg/ml). Three years later MMF was discontinued with the patient still being in drug free remission.

### Case report 3

In 1998 a 7 year old Caucasian boy (Turkish) was diagnosed with systemic lupus erythematosus (SLE), who initially presented with arthralgia, fatigue and malar rash. Laboratory work-up showed an elevated ESR and a normal CRP. Positive anti-dsDNA antibodies and a proteinuria (750 mg/24 h, normal 300 mg) were also found. Renal biopsy proved lupus nephritis (WHO Grade IV). No other organs were involved at that time. Treatment with i.v. MP pulses (1 g/day for 3 days) and p.o. AZA (3 mg/kg/day) was initiated, leading to reduction of the proteinuria (350 mg/24 h) and normalization of the ESR whereas anti-dsDNA antibodies persisted. Due to incompliance, the patient was only inconsistently seen by a physician. Three years after the initial presentation the boy was admitted to the Intensive Care Unit with loss of consciousness and seizures. The MRI of the brain showed subcortical hyperintense lesions, which were regarded as SLE- related cerebral disease. Blood pressure was not elevated and a hypertensive encephalopathy was not considered as a possible cause for the cerebral changes seen on MRI. The elevated ESR and the normal CRP as well as the high levels of anti-dsDNA antibodies, were also supporting the relation to SLE. Intravenous MP pulses (1 g/day for 3 days), plasmapheresis and i.v. CYC (750 mg/m^2 ^in a single dose) (Table 1) showed no effect over the period of two weeks, neither clinically nor serologically. At this point the decision for the experimental use of antiCD20 antibody-treatment was made and one single RTX dose was given. The patient slowly regained consciousness over further two weeks and inflammatory parameters normalized (there are no records on anti-dsDNA antibody levels). Kidney function, though, did not improve during the next two years and the disease progressed to end-stage renal failure (creatinine 8 mg/dl, normal 1.2) requiring hemodialysis. Six years later signs and symptoms of SLE were not present and single kidney transplantation was performed. At that time anti-dsDNA antibodies were not present.

### Case report 4

In 1998 a 13 year old Caucasian girl (Russian), who was diagnosed with SLE and later with myasthenia gravis (MG), presented initially with pain and diffuse swelling of the left leg. Doppler-ultrasonography was performed and revealed deep venous thrombosis (DVT). The diagnosis was supported by elevation of the d-dimers (highest values 4 μg/ml, normal up to 0.5) and therapy with low molecular heparin was initiated. On further questioning she reported the following symptoms which she had been experiencing over the last three months: recurrent fever attacks, increased sweating, fatigue, weight loss (5 kg) and recurrent malar rash, which was not present at admission. The laboratory work-up showed an elevated ESR, a normal CRP and elevated anti-dsDNA antibodies (anti-cardiolipin antibodies were negative). Apart from positive d-dimers (due to the current thrombosis), other coagulation parameters of the thrombophilic work-up were normal. Treatment with AZA (3 mg/kg/day) was initiated (Table 1). Oral PREDN (2 mg/kg/day) was administered as bridging drug. Symptoms gradually disappeared and laboratory parameters including anti-dsDNA antibodies normalized over the period of one year, leading to remission of SLE.

Another year later, the patient complained of increasing fatigue and excertional weakness. Examination showed inability to hold raised arms and legs for more than 30 seconds and also fasciculations of the tongue, diplopic images, which were impairing the vision and nasal speech. Laboratory parameters (ESR, CRP, liver and kidney function tests) including muscle enzymes were normal. Anti-acetylcholine receptor (AChR) antibodies were elevated and myasthenia gravis (MG) was diagnosed. The girl was treated initially with i.v. MP pulses (1 g/day for 3 days), p.o. PREDN (2 mg/kg/day) and AZA (3 mg/kg/day), which were insufficient to control disease activity of MG (SLE was still in remission), with persistent loss of muscle strength and impairment of the ability to perform self-care during the next months. Medication was switched to MMF (1-2 g/day - depending on MMF blood levels; attempted: 3.5-4.5 μg/ml) and oral PREDN was continued in various doses, which were adapted to the disease activity. Pyridostigmine was administered during the entire treatment period (Table 1). Under this medication the patient regained her previous muscle strength allowing her a normal daily life, which period lasted for approximately three years. After that time she presented with symptoms of a new MG flare -impossibility to raise arms and walk properly, diplopia and nasal speech. No clinical or laboratory signs for active SLE disease were found. In order to achieve control of MG six i.v. CYC pulses (750 mg/m^2 ^/every 4 weeks) (Table 1) were administered. Oral PREDN (various doses depending on the disease activity of MG, up to 2 mg/kg/day) and pyridostigmine (various doses adapted to muscle weakness) were continued as concomitant medication. Within the first two months after the start of CYC treatment muscle strength improved and the patient was able to walk again and to perform self care with minor difficulties. Another severe flare of MG (SLE was continuously in remission) occurred seven months after the end of the treatment with CYC. The aggravating muscular weakness could not be controlled by the medication (including i.v. MP pulses, i.v. CYC at doses previously described and plasmapheresis) and treatment with RTX was initiated (Table 1). Despite two RTX courses (Table 1, Figure 1), which were administered six months apart, there was no clinical effect on MG symptoms and further disease progression with continuous loss of muscle strength was noted. Medication including i.v. MP pulses (1 g/day for 3 days), p.o. PREDN (2 mg/kg/day), AZA (3 mg/kg/day) and plasmapheresis did not influence the progressive disease course and ASCT was performed. During the first weeks after ASCT muscle weakness slowly improved allowing the patient to walk again a few steps.Three months after ASCT the improvement was interrupted by another flare of MG with increasing fatigue, impossibility to walk and rising AChR antibodies. A third RTX course (after ASCT, Figure 1) was applied, leading to complete remission of MG symptoms for more than 18 months now.

During the first RTX course only one episode of a mild bronchitis occurred in that patient, which was successfully handled with oral antibiotics in the outpatient clinic.

### Case report 5

In 1991 a 3 year old Caucasian girl (Slovenian) was diagnosed with multiple sclerosis (MS). She initially presented with a slowly developing left sided hemiparesis and bilateral vision impairment, which had occurred within less than a month. Based on clinical presentation, elevated cerebro-spinal fluid (CSF) total protein and positive test for oligoclonal bands in the CSF multiple sclerosis (MS) was diagnosed. Treatment included various drugs including 12 i.v. CYC pulses (750 mg/m^2 ^/every 4 weeks) (Table 1). Despite treatment disease flared several times over the next years leading to almost complete visual loss. Repeated MRIs of the brain also showed areas of persistent gadolinium enhancement suggesting active disease. Due to the relapsing/progressive disease course at the age of 12 years treatment with β-interferon was initiated, which led to clinical and MRI remission over the next five years.

At the age of 17 years, the patient presented with malar rash, fatigue and arthritis in both knees. Laboratory workup showed an elevated ESR, high levels of anti-dsDNA antibodies, slightly reduced complement factors C3 and C4, which were suggestive for SLE. Supposing causality between treatment with β-interferon and drug induced SLE, β-interferon was discontinued and the patient received two i.v. MP pulses (1 g/day for 3 days) and AZA (3 mg/kg/day) (non-steroidal anti-inflammatory drugs were applied for the arthritis) (Table 1) but without any effect, neither clinically nor for the serological parameters.

Further i.v. MP pulses and p.o. PREDN (2 mg/kg/day) were administered, yet without response concerning SLE symptoms (MS was still in remission). The patient did not respond to steroids and also discontinuation of β-interferon did not improve the pathologic condition, therefore de novo SLE was supposed. Further therapeutic considerations included treatment with CYC. The patient had already received more than 25 g CYC in her life and with respect to CYC- related longterm side effects RTX was used (Table 1, Figure 1). SLE disease activity decreased over the next three months: arthritis subsided, malar rash was only slightly visible, ESR normalized, C3 and C4 normalized, although anti-dsDNA antibodies persisted. On a regular check-up three months later renal insufficiency was noticed as reflected by reduction of the creatinine clearance (60 ml/min; normal range 80-120) and a slight elevation of the total urine protein (500 mg in the 24 hours urine collection, normal below 300 mg). Renal biopsy indicated WHO grade V nephritis. Treatment with p.o. MMF (2-3 g/day -depending on MMF blood levels; attempted: 3.5-4.5 μg/ml) was initiated, which had no effect on the progressive renal involvement over the next seven months leading to further reduction of the creatinine clearance (lowest level at 36 ml/min). The latter developed despite effective B-cell depletion (Figure 1). Since the patient had already received multiple i.v. CYC pulses ASCT was performed. Within the first four months after ASCT malar rash vanished entirely, creatinine clearance normalized (95 ml/min) and proteinuria disappeared, anti-dsDNA antibodies were not detectable. Two months later, the patient developed autoimmune hemolytic anemia and thrombocytopenia, which was considered as Evans syndrome. Other SLE symptoms (clinically or in the laboratory work-up) were not present. Another two single RTX doses had no effect on this aspect of the disease and the patient required high doses of i.v. MP (1 g/day for 3 days) and p.o. PREDN (2 mg/kg/day) as well as repeated erythrocyte and platelet transfusions. The condition deteriorated continuously and she died of multiple organ failure due to severe uncontrollable bleeding and hemolysis (Evans syndrome).

### Immunoglobulin levels after treatment with rituximab and B-cell reoccurrence

All patients had normal immunoglobulins before treatment. In patient 1 (JDM), IgG, IgM and IgA-levels were temporarily (over 6 months) reduced below normal range without occurrence of infection. Patient 4 (SLE/MG) had normal levels of all three immunoglobulin types during the first and second RTX course. After the third course (after ASCT) all three immunoglobulines dropped below normal ranges, but in this case the effect on immunoglobulin production cannot be assigned with certainty to RTX, ASCT or both. All other patients had normal immunoglobulin levels after RTX.

The median time for return of peripheral B-cells was 7.3 months (Table 1, Figure 1), although in patients 3-5 the influence of ASCT on B-cell production has to be taken into account.

## Discussion

### Efficacy

Efficacy of RTX was variable in our patients. Patient 2 (WG) achieved remission (both clinically and serologically) after only one RTX course, subsequently sustained with MMF, which was tapered after two years of treatment without disease recurrence.

RTX has been effective for the treatment of pediatric patients with SLE [5,6]. In patient 3 (SLE) cerebral disease was regarded as SLE-related and probably the combination of RTX with immunosuppressive drugs was effective to control cerebral disease [4]. Despite that therapy, kidney disease progressed to end-stage renal insufficiency, requiring haemodialysis and kidney transplantation later on. Similarly, in patient 5 (MS/SLE) kidney disease was progressive despite RTX and kidney function recovered only after ASCT. After discontinuation of β-interferon MS was still inactive in that patient, which might be also related to the treatment with RTX [10], but is most probably the result of the combination treatment with immunosuppressive drugs and ASCT. This patient developed Evans syndrome after ASCT, which was regarded as SLE associated and died of its complications. With respect to renal disease both cases are in contrast with published data on successful application of RTX in children with SLE related kidney disease [5,6].

RTX has also been effective for the treatment of JDM [7]. In patient 1 (JDM) RTX only had a short lasting effect on the JDM progression. It has to be mentioned that this patient had a severe disease course and RTX was considered only late during the treatment where disease progressed despite RTX. The patient achieved a complete drug-free remission after the ASCT.

In patient 4 (SLE/MG) RTX only had a minimal, short lasting effect on the disease progression of MG (SLE was inactive) before ASCT. Interestingly several months after ASCT another MG flare occurred and RTX was applied. This approach led to a complete remission in that patient. In this case treatment success should probably be regarded as a summary effect of both therapies.

### Tolerability and safety

In our five patients, RTX was well tolerated. In contrast to the published literature we could not observe infusion related reactions or serum sickness [5], which is probably due to the anti-allergic pre-treatment prior to every RTX-infusion. Although infections have been related to RTX by different authors [8,9], in our cohort only one episode of a mild pulmonary infection occurred and was successfully treated with oral antibiotics.

### Immunoglobulins and B-cell depletion

A transient reduction of the levels of all three immunoglobulin subtypes was found in two of our patients: patient 1 (JDM) and patient 4 (SLE/MG). Decrease in immunoglobulin levels was not associated with more frequent infections and IVIG substitution was not undertaken. Nevertheless, normal immunoglobulin levels in B-cell depleted patients probably do not imply a normal response to infection. Therefore in case of recurrent or severe infections monitoring of all three immunoglobulin types should be carried out and IVIG substitution considered.

In the available literature immunoglobulins were reduced in some of the patients, though none of them required immunoglobulin substitution due to these low levels [6-8].

B-cell depletion lasted over a median time of 7.3 months in our patient cohort without any clear correlation between the B-cell depletion, B-cell recurrence, improvement/remission and disease flares (Table 1 and Figure 1), a phenomenon already described in the literature [11].

## Conclusion

Children with refractory autoimmune disorders often require treatment with various combinations of immunosuppressants including high doses of steroids and cyclophosphamide. These drugs may have significant side effects and toxicity, especially when given over longer periods of time. Representing a new therapeutic option with a potentially lower rate of adverse events, rituximab may be an important alternative and should be considered alone or in combination treatment.

## Abbreviations

AChR: Anti-acetylcholine receptor antibodies; ALD: Aldolase; anti-dsDNA: Anti doublestranded desoxy-ribonucleotide acid antibodies; ASCT: Autologous stem cell transplantation; AZA: Azathioprine; c-ANCA: proteinase-3 anti-neutrophil cytoplasmatic antibodies; CK: Creatine kinase; CRP: C-reactive protein; CSA: cyclosporine A; CSF: Cerebro-spinal fluid; CYC: cyclophosphamide; DVT: Deep venous thrombosis; ESR: Erythrocyte sedimentation rate; i.v.: intravenous; IVIG: intravenous immunoglobulins; JDM: Juvenile dermatomyositis; MG: Myasthenia gravis; MMF: Mycophenolate mofetil; MP: Methylprednisolone; MRI: Magnetic resonance imaging; MS: Multiple sclerosis; MTX: Methotrexate; NSAIDs: Non-steroidal anti-inflammatory drugs; p.o.: Oral (per os); PREDN: Prednisolone; RA: Rheumatoid arthritis; RTX: rituximab; SLE: Systemic lupus erythematosus; WG: Wegener's granulomatosis.

## Consent

Written informed consent for publication of this case series was obtained from the patients/patients' parents. A copy of the written consent is available for review by the Editor-in-Chief of this journal.

## Competing interests

The authors declare that they have no competing interests.

## Authors' contributions

NT took part in the interpretation of the data of all patients and contributed to the writing and revision of the entire manuscript. JKD analyzed and interpreted the data with respect to JDM and Wegener granulomatosis and contributed to writing the manuscript. IK and JS analyzed and interpreted the data of the patients with SLE and were major contributors in writing and critically revising the.manuscript. All authors read and approved the final version of the manuscript.
